# Bayesian emulation and history matching of JUNE

**DOI:** 10.1098/rsta.2022.0039

**Published:** 2022-10-03

**Authors:** I. Vernon, J. Owen, J. Aylett-Bullock, C. Cuesta-Lazaro, J. Frawley, A. Quera-Bofarull, A. Sedgewick, D. Shi, H. Truong, M. Turner, J. Walker, T. Caulfield, K. Fong, F. Krauss

**Affiliations:** ^1^ Institute for Data Science, Durham University, Durham DH13LE, UK; ^2^ Department of Mathematical Sciences, Durham University, Durham DH13LE, UK; ^3^ Institute for Particle Physics Phenomenology, Durham University, Durham DH13LE, UK; ^4^ Institute for Computational Cosmology, Durham University, Durham DH13LE, UK; ^5^ Advanced Research Computing, Durham University, Durham DH13LE, UK; ^6^ Centre for Extragalactic Astronomy, Durham University, Durham DH13LE, UK; ^7^ Department of Computer Science, Durham University, Durham DH13LE, UK; ^8^ Department of Science, Technology, Engineering and Public Policy, University College London, London WC1E6BT, UK; ^9^ Department of Anaesthesia, University College London Hospital, London NW12BU, UK

**Keywords:** disease models, Bayes linear, emulation, calibration, history matching

## Abstract

We analyze JUNE: a detailed model of COVID-19 transmission with high spatial and demographic resolution, developed as part of the RAMP initiative. JUNE requires substantial computational resources to evaluate, making model calibration and general uncertainty analysis extremely challenging. We describe and employ the uncertainty quantification approaches of Bayes linear emulation and history matching to mimic JUNE and to perform a global parameter search, hence identifying regions of parameter space that produce acceptable matches to observed data, and demonstrating the capability of such methods.

This article is part of the theme issue ‘Technical challenges of modelling real-life epidemics and examples of overcoming these’.

## 1. Introduction: Bayesian emulation and uncertainty quantification

The COVID-19 pandemic disrupted healthcare systems and caused substantial fatalities around the globe. Various models have been developed to aid decision makers in the assessment of policy options, from simple analytic models up to complex agent-based models (ABMs). JUNE, introduced in [1], is of the latter type, and its high level of demographic and spatial resolution demands substantial computational resources to evaluate. A critical component in the uncertainty analysis, and subsequent use for decision support of a complex epidemiological model such as JUNE, is the process of model calibration: the matching of the model to observed data from the real system.

This process can be extremely challenging, and in many cases, its intractability precludes the full exploitation of sophisticated models, which may otherwise contain nuanced insights into the system of interest. The problem of calibrating a complex and computationally demanding model is not unique to epidemiology and occurs in a wide range of scientific disciplines including cosmology, climate, systems biology, geology and energy systems [2–5]. To solve this problem, an area of Bayesian statistics arose, sometimes referred to as the uncertainty analyses of complex computer models, or to use its more recent (and slightly more general name): the area of uncertainty quantification (UQ) [5–7]. UQ provides a statistical methodology combining a large number of efficient techniques with a set of overarching principles that address how to analyze complex models rigorously, transparently and robustly, for use in scientific investigations, for making real-world predictions and for subsequent decision support. A core goal of this work is to demonstrate the capability of such methods for use with complex epidemiological models. A full analysis of the behaviour of models with a large number of input parameters and possibly several outputs, and their subsequent calibration, encounters the following three major issues:

(i) For complex models, the evaluation time of the model is often so long that an exhaustive exploration of the model’s behaviour over its full input parameter space is infeasible.
(ii) When comparing models to observed real-world data, an adequate statistical description of the link between model and reality, covering all major uncertainties and allowing for the rigorous use of an *imperfect* model, is required.
(iii) When calibrating, the appropriate scientific goal should be to identify *all* locations in input parameter space that lead to acceptable fits between model and observed data, and not just to find the location of a single good match.

We summarize in the next section three UQ methods: (a) Bayes linear emulation, (b) linking models to reality and (c) Bayesian history matching, which address the aforementioned three problems. We then apply these UQ methods to the JUNE model in §3.

## 2. Bayesian emulation and history matching

### (a) Bayes linear emulation

Complex models typically have runtimes that can vary from seconds to days or even weeks, greatly inhibiting full model exploration, calibration, forecasting etc. Many of the techniques in UQ therefore revolve around the construction of Bayesian *emulators*: statistical constructs that mimic the scientific model in question, providing predictions of the model outputs with associated uncertainty, at as yet unevaluated input parameter settings [8]. The emulators provide insight into the model’s core structure and, unlike the models they mimic, are extremely fast to evaluate, typically being several orders of magnitude faster. Hence, they facilitate previously infeasible model exploration and global parameter searches. As an emulator makes predictions that have an associated (input dependent) uncertainty statement, they naturally fit within an overarching Bayesian uncertainty analysis, in which the impact of using an emulator instead of the model, can be understood and quantified. Emulators can be built for deterministic models, stochastic models, multilevel models (composed of models of increasing fidelity) and networks of models, providing a flexible and powerful set of tools to deal with a large class of scientific scenarios. Here, we outline the construction of Bayes Linear emulators, a robust form of emulator, based on a partial specification, which has been successfully employed in several settings [2,5].

We represent a general scientific model as the function f(x). Here, $x=(x_{1},\ldots ,x_{d})$ is a vector composed of all the input parameters. For example, $x_{1}$ may represent an infectivity parameter, $x_{2}$ a social distancing parameter, etc. $f(x)=(f_{1}(x),\ldots ,f_{q}(x))$ is the vector of all model outputs of interest, so, for example, $f_{1}(x)$ may represent the number of people hospitalized in England on a particular day, $f_{2}(x)$ may represent the number of deaths on that day, all as a function of the inputs x. We denote the general component of f(x) as $f_{i}(x),$ where the index i will cycle through the full list of outputs of interest, for example, in the application to JUNE in §3, i cycles through the set i∈{type,region,time}. We anticipate that, due to limited computational resources, we will only be able to evaluate the model at a finite (and possibly small) number of input parameter locations $x^{\left(1\right)},x^{\left(2\right)},\ldots ,x^{\left(n\right)}$ giving rise to model outputs $D_{i}={\left(f_{i}(x^{\left(1\right)}),f_{i}(x^{\left(2\right)}),\ldots ,f_{i}(x^{\left(n\right)})\right)}^{T}$, where i=1,…,q, and ‘T’ denotes the transpose. Therefore, at a new unevaluated input location, x, even say for a deterministic (i.e. repeatable) model, we will still be uncertain about the output value of f(x), as we will be for the majority of the input space. We take a subjective Bayesian view and treat the unknown f(x) as a random quantity and construct an emulator that represents our beliefs about possible reasonable forms that this function f(x) could take. A popular emulator form for each output $f_{i}(x)$ is as follows [2]:

2.1 $$f_{i}(x)=\sum_{j}b_{ij}g_{ij}(x_{A_{i}})+u_{i}(x_{A_{i}})+w_{i}(x),$$

where we have selected a subset of the inputs, x, known as the active variables, $x_{A_{i}}$, that are most influential for output $f_{i}(x)$. The first term on the right-hand side of equation (2.1) is a regression term, where $g_{ij}$ are appropriately selected known deterministic functions of $x_{A_{i}}$, a common choice being low-order polynomials, and $b_{ij}$ are unknown scalar regression coefficients. The second term, $u_{i}(x_{A_{i}})$, is a weakly second-order stationary process over $x_{A_{i}}$, for which we only need to specify its second-order structure, choosing $E[u_{i}(x_{A_{i}})]=0$ and utilizing an appropriate covariance function: a classic example suitable for smooth functions is the squared exponential:

2.2 $$\mathrm{Cov}(u_{i}(x_{A_{i}}),u_{i}(x_{A_{i}}^{\prime }))=\sigma_{u_{i}}^{2}\exp\left\{-\frac{||x_{A_{i}}-x_{A_{i}}^{\prime }|{|}^{2}}{\theta_{i}^{2}}\right\},$$

where $\sigma_{u_{i}}^{2}$ and $\theta_{i}$ are the variance and correlation length of $u_{i}(x_{A_{i}}),$ respectively, which may be specified *a priori* [2], or fitted using, e.g. MLE or MAP [4]. This simple covariance function may be enough, especially if the emulators regression term captures much of the model’s behaviour; however, if not, various extensions are available, e.g. individual correlation lengths $\theta_{i}^{j}$ for each input $x_{A_{i}}^{j}$ [9]. The third term, $w_{i}(x)$, is a white noise process uncorrelated with $b_{ij}$, $u_{i}(x_{A_{i}})$, and itself such that

2.3 $$\mathrm{Cov}(w_{i}(x),w_{i}(x^{\prime }))=\left\{ \begin{array}{cl} \sigma_{w_{i}}^{2} & \mathrm{if}\;x=x^{\prime }\\ 0 & \mathrm{otherwise} \end{array}\right.,$$

with expectation zero, and $\mathrm{Var}(w_{i}(x))=\sigma_{w_{i}}^{2}$. $w_{i}(x)$ represents the effects of the remaining inactive inputs not included in the list of $x_{A_{i}}$ and formally facilitates a type of dimensional reduction [2].

The emulator form, as represented by equation (2.1), has various desirable features and exploits our beliefs about the general anticipated behaviour of physically realistic models. The regression term, $\sum_{j}b_{ij}g_{ij}(x_{A_{i}})$, attempts to mimic the large-scale global behaviour of the function $f_{i}(x)$: often substantial in physical models. The second term, $u_{i}(x_{A_{i}})$, the weakly stationary process, mimics the local behaviour of $f_{i}(x)$, again exploiting concepts of smoothness of either $f_{i}(x)$ or attributes of $f_{i}(x)$ (if, say, $f_{i}(x)$ is stochastic). Such terms are highly versatile and can fit a large class of models; however, they require a sufficient density of runs to be suitably informed (regulated by the correlation length parameters $\theta_{i}$). In the literature, there is sometimes an over-reliance on similar Gaussian process style terms and a neglect of the regression terms, which may be unwise, as GPs of this form are typically capable of capturing the broad global behaviour, or the more complex local behaviour, but rarely both. We deliberately use the regression terms for the global structure and utilize the $u_{i}(x_{A_{i}})$ to capture the local behaviour.

We can select the list of active inputs, $x_{A_{i}}$, using various statistical techniques. For example, these could consist of classical linear model fitting criteria such as AIC or BIC, which have the benefit of speed and reasonable accuracy when applied to appropriate (nonlinear) sets of regression functions $g_{ij}$ [2], or approaches such as automatic relevance determination [9], which can give increased accuracy provided the assumed form of the covariance function is suitable. In addition, we would also seek to incorporate expert knowledge of the model into the active input selection process, either by directly incorporating 'known' active inputs or by using a more nuanced Bayesian approach, of particular importance for expensive models. A list of p active inputs for a particular physical output, $f_{i}(x)$, means that we have notably reduced the input dimensionality from d to p, which can result in large efficiency gains in subsequent calculations. The small remaining effect of the inactive inputs is not ignored, but is captured by the third term $w_{i}(x)$ in equation (2.1), whose variance $\sigma_{w_{i}}^{2}$ represents the added uncertainty induced by the dimensional reduction.

#### (i) What to emulate

A major issue when emulating complex models is the choice of the set of attributes/outputs of the model to emulate. For example, often an objective function describing the mismatch between model and data has been emulated (e.g. a simple chi-squared metric, or a more complex likelihood function). However, despite being deceptively simple having just a single output, the objective function typically has a complex form, as it depends on the union of all active inputs and possesses numerous local maxima/minima [2], rendering this an inefficient strategy. Instead, we prefer to emulate the physical outputs of the model directly, as these tend to have (a) a smaller list of active inputs per output allowing a nuanced and sometimes substantial dimensional reduction tailored to each individual output, and (b) a simpler functional dependence on the input parameters that is often well represented by the regression terms in the emulator. Further choices are required when emulating stochastic models, where we can choose to emulate summaries of outputs of interest such as the mean, the variance or quantiles if required, possibly conditioning on key events such as epidemic take-off, and extend for example to covariance structures between groups of outputs if needed. In these cases, the role of $w_{i}(x)$ is extended to also incorporate the uncertainties induced by using estimates from finite samples [10] or to employ full variance emulation as in ref. [11].

#### (ii) Designing batches of model evaluations

We begin by specifying the region of input space of interest, typically a d-dimensional hypercube, and denote this $X_{0}\subset R^{d}$. We then design a set of ‘space filling’ runs over $X_{0}$, constructed to be well spread out, without any large holes. For example, we may use a maximin Latin hypercube design, an approximately orthogonal design, also desirable for emulator construction [12,13].

#### (iii) Updating the emulator

We then update our prior emulator structure given by equation (2.1) with the information from the set of model runs using our favourite statistical tools. Specifically, we would prefer a fully probabilistic Bayesian approach if we required full probability distributions on all emulated outputs, $f_{i}(x)$ [14], and were we prepared to specify full joint probabilistic priors.

However, in most cases, our preferred choice is to use Bayes Linear methods, a more tractable version of Bayesian statistics, which requires a far simpler prior specification and analysis [15,16]. It deals purely with expectations, variances and covariances of all uncertain quantities of interest, and uses the following Bayes linear update equations, derived from foundational arguments [16], to adjust our beliefs in the light of new data. When emulating the ith output $f_{i}(x)$ of a complex model, where we had performed an initial batch of n runs giving a vector of model output values $D_{i}={\left(f_{i}(x^{\left(1\right)}),f_{i}(x^{\left(2\right)}),\ldots ,f_{i}(x^{\left(n\right)})\right)}^{T}$, we obtain the adjusted expectation, $E_{D_{i}}(f_{i}(x))$, and adjusted variance, ${\mathrm{Var}}_{D_{i}}(f_{i}(x))$, for $f_{i}(x)$ at new input point x using:

2.4 $$E_{D_{i}}(f_{i}(x))=E(f_{i}(x))+\mathrm{Cov}(f_{i}(x),D_{i})\mathrm{Var}{\left(D_{i}\right)}^{-1}(D_{i}-E(D_{i}))$$

and

2.5 $${\mathrm{Var}}_{D_{i}}(f_{i}(x))=\mathrm{Var}(f_{i}(x))-\mathrm{Cov}(f_{i}(x),D_{i})\mathrm{Var}{\left(D_{i}\right)}^{-1}\mathrm{Cov}(D_{i},f_{i}(x)).$$

**Figure 1.**
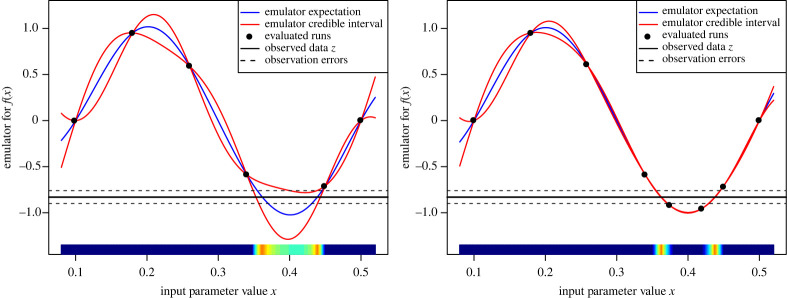
An emulator of a one-dimensional toy model, where f(x)=sin⁡(2π(x−0.1)/0.4), for the first wave/iteration, using just six runs (left panel), and for the second wave, using two additional runs (right panel). The emulator’s expectation $E_{D}[f(x)]$ and credible intervals $E_{D}[f(x)]\pm 3\sqrt{{\mathrm{Var}}_{D_{i}}(f_{i}(x))}$ are given by the blue and red lines, respectively, with the observed data z that we wish to match to as the black horizontal line (with errors). The implausibility I(x) is represented by the coloured bar along the x-axis, with dark blue implying I(x)>3, light blue 2.5<I(x)<3 and yellow (I(x)<1). (Online version in colour.)

All quantities on the right-hand side of equations (2.4) and (2.5) can be calculated from equations (2.1) and (2.2) combined with prior specifications for $E(b_{ij})$, $\mathrm{Var}(b_{ij})$, $\sigma_{u_{i}}^{2}$, $\sigma_{w_{i}}^{2}$ and $\theta_{i}$. $E_{D_{i}}(f_{i}(x))$ and ${\mathrm{Var}}_{D_{i}}(f_{i}(x))$ provide a prediction for $f_{i}(x)$ with associated uncertainty and are used directly in the implausibility measures used for the global parameter searches described in §2c. Note that multivariate versions of equations (2.4) and (2.5) are available. In addition, we may make certain pragmatic choices in the emulator construction process, for example, while we typically keep the regression coefficients $b_{ij}$ uncertain, we may directly specify $\sigma_{u_{i}}^{2}$, $\sigma_{w_{i}}^{2}$ and $\theta_{i}$ *a priori*, or use suitable plugin estimates as described in ref. [2]. We can test the emulators using a series of diagnostics, for example checking their prediction accuracy over a new batch of runs [17]. An example of a one-dimensional emulator is given in figure 1, cf. ref. [8] for an introduction and refs. [2,14,18] for details. The above Bayes linear emulation framework is fully implemented in the ‘hmer’ R package [19].

### (b) Assessing uncertainties: linking the model to the real world

Most epidemiology models are developed to help explain, understand and predict the behaviour of the corresponding real world system of interest, typically in terms of the progression through a population of an infectious disease. An essential part of determining whether such a model is adequate for this task is the comparison of the model with data formed from observations of the real system. However, this comparison involves several uncertainties that must be accounted for to provide a meaningful definition of an ‘acceptable’ match between a model run and the observed data. Hence, it is vital to define a clear statistical model describing the difference between the epidemiological model, f(x), and the observed data denoted as the vector z. While more complex statistical models are available [20], here, we describe a simple but powerful version that has been successfully used in a variety of scientific disciplines, for example climate, cosmology, oil reservoirs, epidemiology and systems biology [2–5,10,21].

The most familiar source of uncertainty is observational or experimental. We represent the features of interest of the real system as a vector of uncertain quantities, y, which will be measured imperfectly involving a vector of errors e, to give the vector of observations, z, as follows:

2.6 z=y+e.

We represent the errors as additive here, but could use a more complex form if necessary. Depending on the scientific context, we then make judgements about the relationship between y and e, e.g. a common specification [2] is to judge the errors e to be independent from y, with expectation, $E(e)=\mu_{e}$ and $\mathrm{Var}(e)=\Sigma_{e}$, a q×q covariance matrix. Setting $\mu_{e}=0$ corresponds to the judgement that the observations were unbiased, and setting $\Sigma_{e}=\mathrm{diag}(\sigma_{e,1}^{2},\ldots ,\sigma_{e,q}^{2})$, that is a diagonal matrix, corresponds to uncorrelated observation errors, etc.

A critical feature that we must incorporate is the difference between the epidemiological model, f(x), of the system and the real system, y, itself. We represent this difference between model and reality using a *structural model discrepancy* term. First, we note that even were we to evaluate the model, f(x), at its best possible choice of input, $x^{*}$, the output, $f(x^{*})$, would still not be in agreement with the real epidemiological system value y, due to the many simplifications and approximations inherent to the model; therefore,

2.7 $$y=f(x^{*})+\epsilon ,$$

where ϵ is the structural model discrepancy: a vector of uncertain quantities that directly represents the difference between the model and the real system. Note that we are still treating y, f, $x^{*}$ and ϵ as vectors of random quantities. Now we have to make judgements about their relationships: a simple and popular specification [2,5] would be to judge that ϵ is independent of $f(x^{*})$, $x^{*}$ and e, with E(ϵ)=0. In the case of a single output, we would then specify $\mathrm{Var}(\epsilon )=\sigma_{\epsilon }^{2}$. However, for the full case of q outputs, we may specify $\mathrm{Var}(\epsilon )=\Sigma_{\epsilon }$, a q×q covariance matrix. $\Sigma_{\epsilon }$ may have intricate structure possessing non-zero covariances between components of ϵ, to capture the heavily correlated deficiencies of the model outputs. Various structures for $\Sigma_{\epsilon }$ of increasing complexity are available [2,5,14], along with methods for their specification [2,22]. Note that typically the form of $\Sigma_{\epsilon }$ is very different from $\Sigma_{e}$.

While the inclusion of the structural model discrepancy is unfamiliar to most modellers, it is now of standard practice in the UQ literature for analyzing complex but imperfect models [14,18,23,24]. It facilitates a richer analysis whereby we can incorporate our necessarily uncertain knowledge of the model’s deficiencies to improve our modelling of reality y. Its inclusion can prevent over-fitting when calibrating and also reduces both bias and overconfidence when predicting. It is also vital when combining the results of several models.

### (c) Bayesian history matching

Due to their fast evaluation speed, emulators can be used in a variety of UQ calculations that would be otherwise infeasible. One of the most important is the problem of performing global parameter searches. Here, we outline a powerful iterative emulator-based global search method known as history matching (HM), which has been successfully employed in a variety of scientific disciplines [2,3,5,10]. HM is designed to answer the questions:

(i) Are there any input parameter settings that lead to acceptable matches between the model output and observed data?
(ii) If so, what is the full set X that contains all such input parameter settings?

Note the emphasis on finding *all* such acceptable matches: optimizing to find a single good fit is not adequate for assessing the impact of parametric uncertainty, nor for making predictions.

HM proceeds iteratively by ruling out regions of input parameter space that can be discarded from further investigation based on *implausibility measures* [5]. For an unexplored input parameter, x, we can ask how far would the emulator’s expected value for the individual function output, $f_{i}(x)$, be from the corresponding observed value, $z_{i}$, before we would deem it highly unlikely for $f_{i}(x)$ to give an acceptable match were we to actually evaluate the function at x. The implausibility measure, $I_{i}(x)$, captures this concept, and for an individual, output is given by the distance $E_{D_{i}}(f_{i}(x))-z_{i}$ between emulator expectation and observed data, standardized by all relevant uncertainties,

2.8 $$I_{i}^{2}(x)=\frac{{\left(E_{D_{i}}(f_{i}(x))-z_{i}\right)}^{2}}{{\mathrm{Var}}_{D_{i}}(f_{i}(x))+\mathrm{Var}(\epsilon_{i})+\mathrm{Var}(e_{i})}.$$

Here, ${\mathrm{Var}}_{D_{i}}(f_{i}(x))$ is the emulator variance, $\mathrm{Var}(\epsilon_{i})$ is the variance of the model discrepancy and $\mathrm{Var}(e_{i})$ is the variance of the observational error, a direct consequence of equations (2.6) and (2.7). See also figure 1 (the x-axis) for a depiction of I(x).

A large value of $I_{i}(x)$ for a particular x implies that we would be unlikely to obtain an acceptable match between $f_{i}(x)$ and $z_{i}$ were we to run the model at x. Hence, we can discard the input, x, from the parameter search if $I_{i}(x)>c$, for some cutoff, c, which is often chosen by appealing to Pukelsheim’s 3-sigma rule [25], a very general and powerful result, which states that for *any* continuous, unimodal distribution, 95% of its probability must lie within ±3σ, regardless of asymmetry or skew, suggesting that a choice of c=3 may be reasonable [2]. This is the simplest univariate form, but we can combine implausibility measures from several outputs using say $I_{M}(x)=\max_{i\in Q}I_{i}(x)$ for some set Q, or employ more complex multivariate forms [2].

Before performing the kth HM iteration, we define the current set of non-implausible input points as $X_{k}$ and the set of outputs that we considered for emulation in the previous wave as $Q_{k-1}$. We proceed as follows [4]:

1. Design and evaluate a well chosen set of runs over the current non-implausible space $X_{k}$, e.g. using a maximin Latin hypercube with rejection [2]. Combine these with any non-implausible runs surviving from previous waves.
2. Check if there are new, informative outputs that can now be emulated accurately (that were difficult to emulate in previous waves) and add them to the previous set $Q_{k-1}$, to define $Q_{k}$.
3. Use the runs to construct new, more accurate emulators defined only over the region $X_{k}$ for each output in $Q_{k}$.
4. The implausibility measures $I_{i}(x)$, $i\in Q_{k}$, are then recalculated over $X_{k}$, using the new emulators.
5. Cutoffs are imposed on the implausibility measures $I_{i}(x)<c$ and this defines a new, smaller non-implausible volume $X_{k+1}$, which should satisfy $X\subset X_{k+1}\subset X_{k}$.
6. Unless (a) the emulator variances for all outputs of interest are now small in comparison to the other sources of uncertainty due to the model discrepancy and observation errors, or (b) the entire input space has been deemed implausible, and return to step 1.
7. If 6 (a) is true, generate as large a number as possible of acceptable runs from the final non-implausible volume X, sampled depending on scientific goal.

The history matching approach is powerful for several reasons: (a) while reducing the volume of the non-implausible region, we expect the function f(x) to become smoother, and hence to be more accurately approximated by the regression part of the emulator, $b_{ij}g_{ij}(x_{A_{i}})$. (b) At each new HM iteration, we will have a higher density of points and hence the second term, $u_{i}(x_{A_{i}})$, in the emulator should be more effective, as it depends on proximity to the nearest runs. (c) In later iterations, the previously strongly dominant active inputs from early waves will have their effects curtailed, and hence, it will be easier to select additional active inputs, unnoticed before. (d) There may be several outputs that may be difficult to emulate in early iterations (perhaps because of their erratic behaviour in uninteresting parts of the input space) but simple to emulate in later waves once we have restricted the input space to a much smaller and more epidemiologically realistic region. See ref. [4] for further discussions comparing HM with Bayesian MCMC and ABC, ref. [26] for a direct comparison with ABC and the R package ‘hmer’ [19] for full implementation of the HM algorithm. We now apply these methods to the JUNE model.

## 3. Application of emulation and history matching to JUNE

### (a) The JUNE model

JUNE [1] is an ABM that describes the spread of an infectious disease through large synthetic populations. Originally designed to simulate the circulation of COVID-19 through the English population, JUNE has also been adapted to capture the populations of Cox’s Bazaar [27], a refugee camp in Bangladesh, and of Rhineland-Palatinate [28], one of Germany’s federal states. JUNE’s description of the epidemic spread rests on four areas:

— the construction of a realistic synthetic population that reflects, as accurately as possible, the population demographic and their geographic distribution;
— the simulation of the population sociology, i.e. how the individuals behave: how they spend their time, whom they get into contact with and in which social environment;
— the parameterization of the infection, how it is transmitted from infected to susceptible individuals and impact it has on the health of infected individuals;
— the mitigation of spread and impact of the infection through pharmaceutical and non-pharmaceutical interventions (NPIs) such as social distancing and vaccinations, respectively.

They are discussed in more detail below.

#### (i) Population

JUNE builds its synthetic population based on real or parameterized census data—in the case relevant for this contribution, JUNE constructs the about 55 million residents of England based on the 2011 census data accessible through the NOMIS database provided by the ONS. The data are organized hierarchically, with Output Areas (OAs) the smallest relevant unit, comprising on average about 250 residents with relatively similar socio-economic characteristics. The OAs have a specified geographic location, and their data contain information about age, sex and ethnicity of the area’s residents [29–31] and the composition of the households they live in [32], in about 20 categories.^1^ JUNE uses national averages to correlate age, sex and ethnicity of individuals, which are presented as uncorrelated distributions in the data. In a similar way, information such as the national distributions of age differences of partners [33], and of parents and their children [34], are used to assign the individuals to their households.

As additional static properties of the population, JUNE assigns school-aged children to the nearest age-appropriate school; information about school locations and the age ranges for their students is taken from ref. [35]. Within the schools, the students are grouped into class units of 20–30 individuals and have teachers assigned to them. In a similar way, universities are filled with students—the young adults—and they are grouped into year groups of about 200 students.

The OAs are part of Middle Super Output Areas (MSOAs) with about 12 500 residents and 50 OAs constituting one MSOA. The census data provide information about the sectors of companies within MSOAs and about the distribution of the working population over these sectors, using the Standard Industrial Classification (SIC) scheme [36]. The parameterization of company sizes with national sector-dependent averages allows JUNE to construct an origin-destination matrix for the employees at the level of MSOAs [37]. Information concerning the commuting habits of individuals contained in the census data [38] underpins the construction of simplified virtual public transport networks within JUNE.

#### (ii) Interactions

Having defined the static properties of the synthetic population—where people live, work and study—their daily lives outside work and education are filled with various activities. These activities include shopping, visiting friends and relatives in their homes, frequenting pubs and restaurants, going to the gym or cinema, to name a few. In the absence of any of these leisure activities, people are supposed to stay at home. Surveys performed, e.g. by the Office for National Statistics [39], define the average proportion of time spent with various activities, in dependence on age and sex. These averages are translated into a probabilistic treatment thereby creating a highly flexible and varied daily schedule for JUNE’s virtual individuals.

These schedules are supplemented with contact matrices from PolyMod [40] and the BBC Pandemic Project [41], which indicate the average number of daily contacts—communication or physical—of individuals of age i with individuals of age j in different social settings L, for example home (H), school (S) and work (W). As the contact numbers are presented as population averages, suitable for their deployment in compartment models, they need to be renormalized for the socially more granular IBMs,^2^ resulting in the renormalized overall contact matrices $\chi_{ij}^{\left(L\right)}$ and the corresponding fraction of physical contacts, $\phi_{ij}^{\left(L\right)}$, where L∈{S,H,W}. While this introduces some uncertainty into the modelling of social interactions, the interplay of the synthetic population model with the contact matrices provides a welcome closure test for the self-consistency of the overall model.

For the purpose of fitting to data and the quantification of uncertainties in the model, we assume that the construction of the synthetic population and its interactions is well understood and robustly and well parameterized as it is driven by data of relatively high quality.

#### (iii) Infection

The description of the infection consists of two separate parts. First, the transmission from an infected person i to a susceptible person s needs to be simulated. In JUNE, as in many other models, this is described as a probabilistic process. The infection probability for a susceptible person s with susceptibility $\psi_{s}$ during a time interval from t to t+Δt, spent with a group of individuals g in social context L is given as follows:

3.1 $$\begin{array}{rl} P_{si}(t,t+\Delta t) & \;=1-\exp\left[-\psi_{s}\sum_{i\in g}\int_{t}^{t+\Delta t}dt^{\prime }\;\beta_{si}^{\left(L,g\right)}I_{i}(t^{\prime })\right]\\ & \;\approx 1-\exp\left[-\psi_{s}\Delta t\sum_{i\in g}\beta_{si}^{\left(L,g\right)}I_{i}(t)\right]. \end{array}$$

In the aforementioned equation, $I_{i}(t)$ denotes the time-dependent infectiousness of the infected individual i in group g. In JUNE, it follows a profile given by

3.2 $$I_{i}(\tau )=I_{i\max}\frac{\tau^{a-1}\;e^{-\tau }}{\Gamma (a)},$$

with $\tau =t-t_{0}-t_{\mathrm{inc}}$, and $t_{0}$ is the time of infection of the individual, $t_{\mathrm{inc}}$ is the incubation period and Γ is the gamma function. $t_{\mathrm{inc}}$ is sampled from a normal distribution. The maximal or peak value of infectiousness for individual i is sampled from a log-normal distribution with median exp⁡(μ)=1 and shape parameter σ=0.25, which allows for a long but small tail of highly infectious individuals, which can be connected to super-spreader events. The $\beta_{si}^{\left(L,g\right)}$ in equation (3.1) is the contact intensity between s and i,

3.3 $$\beta_{si}^{\left(L,g\right)}=\beta_{L}\frac{\chi_{si}^{\left(L\right)}}{N_{g}}[1+\phi_{si}^{\left(L\right)}(\alpha -1)],$$

where β are the social location-dependent baseline intensities, $N_{g}$ is the number of individuals in the group setting, normalizing the contact number $\chi_{si}$ and α parameterizes the relative increase in infection probability for the proportion of physical contacts $\phi_{si}$. These parameters, the social-environment dependent β and the universal α, cannot be derived from first principles and must be obtained from fits to available data; they constitute a significant portion of the parameter space in the model and, correspondingly, a significant source of uncertainty.

Once an individual is infected, it takes some time—the incubation period—before they can infect others and some additional time before the onset of symptoms. A large range of input data has been used to derive various symptom trajectories for infected individuals, which in the case of high-income western countries in the global North depends mainly on their age and sex.^3^ In the original formulation of the JUNE model, significant efforts have gone into the quantification of probabilities for different health outcomes in the population, with some emphasis to also capture the health impact of COVID-19 on the highly vulnerable care home residents; we refer the reader to ref. [1] for more details. Here, it should suffice to state that in JUNE asymptomatic and symptomatic trajectories with varying severity have been identified, the latter ranging from mild, flu-like symptoms over admission to regular or intensive-care wards to death in hospital or at residence. Although there are some uncertainties related to this treatment, we usually do not consider them and treat the health outcomes as fixed by data. We seed initial infections based on the number of fatalities 2–3 weeks afterwards, by using the infection-fatality rates obtained from data and encoded in JUNE. The parameter $\alpha_{\mathrm{seedstrength}}$ is an additional factor that modifies the resulting number of initial infections.

#### (iv) Interventions

Since the beginning of the COVID-19 epidemic, the UK government—like many other governments around the world—has employed a wide range of mitigation strategies. At the beginning of the pandemic, these interventions were mainly non-pharmaceutical, and these NPIs ranged from relatively simple strategies at the level of individuals, such as mask wearing and other social distancing measures, to more involved and global strategies such as partial or complete lockdowns, involving school closures and the furloughing of parts of the work force. In JUNE, these measures can easily be modelled: social distancing measures and mask wearing can be described by modifying the β’s in the corresponding social settings by a factor, $M_{L}$, capturing the reduced, but non-zero, transmission probability, while the closure of schools or furloughing of the work force is easily described, based on data [42–45], by keeping the impacted population at home instead of sending them to schools or work. For a more detailed description of the translation of NPIs to the JUNE simulation, we refer the reader to ref. [1].

### (b) Inputs, outputs and initial emulation

Our primary goal is to test if the JUNE model can produce acceptable matches to observed data at the national and regional level, from the first wave of the COVID-19 pandemic and the subsequent summer period. We wish to identify the region of parameter space, X, leading to such acceptable fits, if it exists. We identify a large set of input parameters, x, of interest to search over, primarily composed of interaction intensity parameters at the group level, seeding and quarantine compliance parameters, and social distancing parameters (see [1] for details), and specify ranges for each, given in table 1, which define the initial search space, $X_{0}$. These ranges were chosen to be conservative, informed in part by earlier exploratory runs while also respecting the role each parameter plays in the model, including the time period over which they operate (see [1] for details). A typical full England run of JUNE would take approximately 10 hours to complete on 64 cores (Intel Xeon Skylake) and 128 GB of memory. This substantial computational expense combined with a relatively high-dimensional input parameter space makes a global parameter search extremely challenging and necessitates the use of emulation. While there are several types of data available for the early pandemic, many of these had questions regarding reliability. For example, case data were substantially affected by the limited and evolving availability of COVID-19 tests, while hospital admission data were, especially during the peak of the first wave, collected with understandably varying levels of diligence across trusts. While it would in principle be possible to incorporate such data sets using a detailed statistical model for the measurement errors, e, in equation (2.6) that incorporated under-counting, we instead focus on hospital and total death data [46–49], which although still uncertain due to the precise definition of death with COVID-19, suffers from far fewer issues.

**Table 1.** The input parameters explored in the global parameter search, their type and their ranges that define the search region $X_{0}$.

input parameter $\left(x_{i}\right)$	type	range
$\beta_{\mathrm{pub}}$	location-dependent contact intensity	[0.02,0.6]
$\beta_{\mathrm{grocery}}$	—	[0.02,0.6]
$\beta_{\mathrm{cinema}}$	—	[0.02,0.6]
$\beta_{\mathrm{university}}$	—	[0.02,0.6]
$\beta_{\mathrm{city}\;\mathrm{transport}}$	—	[0.08,0.77]
$\beta_{\mathrm{inter}\;\mathrm{city}\;\mathrm{transport}}$	—	[0.08,1.2]
$\beta_{\mathrm{hospital}}$	—	[0.08,1.2]
$\beta_{\mathrm{care}\;\mathrm{home}}$	—	[0.08,1.2]
$\beta_{\mathrm{company}}$	—	[0.08,1.2]
$\beta_{\mathrm{school}}$	—	[0.08,1.2]
$\beta_{\mathrm{household}}$	—	[0.08,1.2]
$\beta_{\mathrm{carevisits}}$	—	[0.1,10]
$\beta_{\mathrm{householdvisits}}$	—	[0.1,10]
$\alpha_{\mathrm{physical}}$	physical contact factor	[1.8,3]
$\alpha_{\mathrm{seed}\;\mathrm{strength}}$	modifies initial/seeding infections	[0.1,2]
$M_{\mathrm{quarantine}\;\mathrm{household}\;\mathrm{compliance}}$	quarantine compliance	[0.034,0.26]
$M_{\mathrm{socialdistancing}}\;\beta_{\mathrm{factor}}$	social distance (1 week prior to lockdown)	[0.65,0.95]
$M_{\mathrm{sd}3\;\mathrm{random}\;\mathrm{factor}\;\mathrm{all}}$	enhanced social distance (full lockdown)	[0.1,0.5]
$M_{\mathrm{sd}4\;\mathrm{random}\;\mathrm{factor}\;\mathrm{all}}$	social distance (post lockdown, non-leisure)	[0.25,1]
$M_{\mathrm{sd}4\;\mathrm{random}\;\mathrm{factor}\;\mathrm{leisure}}$	social distance (post lockdown, leisure)	[0.25,1]

**Figure 2.**
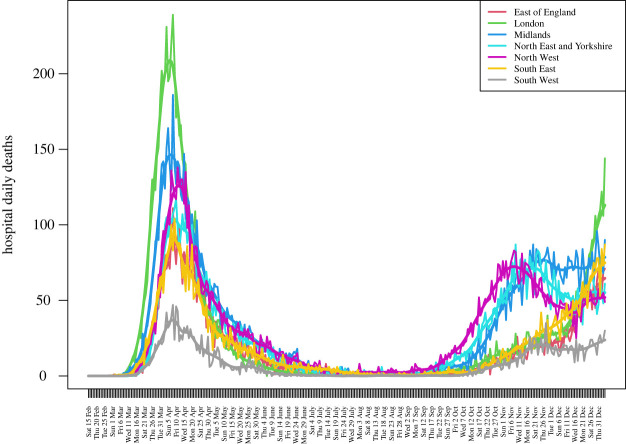
Daily deaths in hospital wards and ICU in 2020, by region. The smoothed version used in the HM is also shown. (Online version in colour.)

We define the primary JUNE outputs of interest to be the hospital deaths and total deaths from 19 March to the end of August 2020, for England and its seven regions: East of England, London, Midlands, North East and Yorkshire, North West, South East and South West. We choose a subset of dates $\left\{t_{1},t_{2},\ldots \right\}$, shown as the vertical dashed lines in figure 4, to emulate. The observed data and JUNE output is noisy, so we smooth them both using a standard kernel smoother (Gaussian kernel, bandwidth 7 days) as we wish to compare the underlying trends, and define the smoothed versions to be the target observed data points $z_{i}$ (shown in figure 2), and the primary JUNE outputs, $f_{i}(x)$. Therefore, i index cycles through elements of the set i∈{type,region,time}, where type labels hospital or total deaths, region labels each of the seven regions of England or England itself and time labels the time points $\left\{t_{1},t_{2},\ldots \right\}$ of interest, given as the dashed lines in figure 4. We specify conservative observation error and model discrepancy variances $\sigma_{e_{i}}^{2}$ and $\sigma_{\epsilon_{i}}^{2}$ for each output as described in ref. [1], by decomposing each into multiplicative and additive components to represent possible systematic biases, in addition to a scaled $\sqrt{n}$ component for the observation error only, to model the noisy count process.

**Figure 3.**
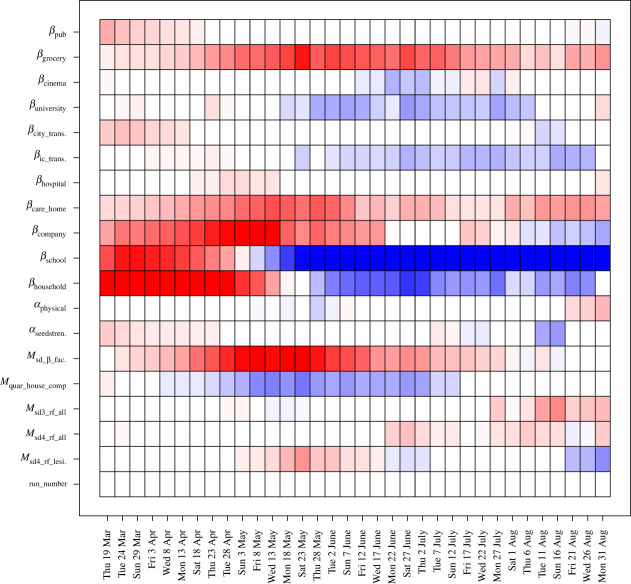
Estimates for the coefficients $b_{ij}$ of the linear terms $g_{ij}(x_{A_{i}})=x_{A_{i}}^{j}$ that are found to feature in the emulators for total deaths in England for the first iteration/wave of runs, where i labels the time point (x-axis) and j labels the inputs (y-axis). Red/blue represents positive/negative dependencies of $f_{i}(x)$ on that input, respectively, standardized as proportions of the largest coefficient for that time point. A finer temporal resolution is used here for added clarity. Note that this plot shows the time-dependent sensitivity of the model to the inputs, but that the actual inputs x do not vary over time. (Online version in colour.)

**Figure 4.**
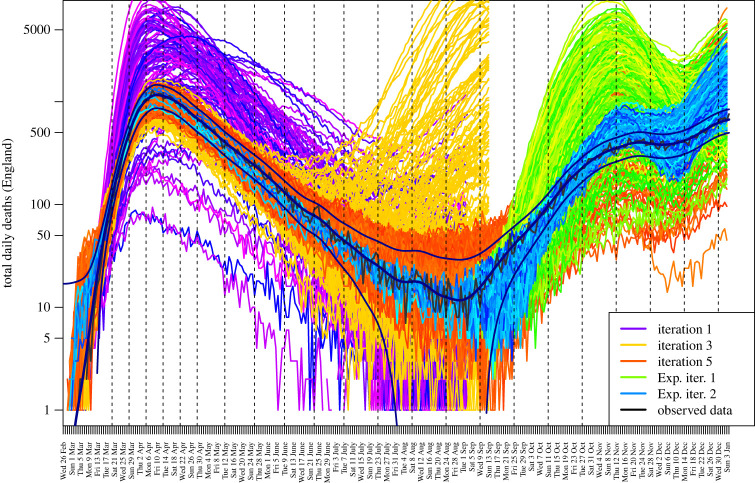
The JUNE output for total daily deaths in England in 2020, for several iterations of the HM process. The smoothed and noisy data, along with the combined uncertainties due to $\sigma_{e}$ and $\sigma_{\epsilon }$, are shown in black. (Online version in colour.)

We design and evaluate a first iteration/wave of 150 runs over the input space, $X_{0}$, using a maximin Latin hypercube design. The outputs of these runs are shown as the purple lines in figure 4. We construct emulators for each output, $f_{i}(x)$, as detailed in §2a (using full quadratic regression terms selected using BIC, and MAP estimates for $\theta_{i}$ [2]). The emulators provide insight into the behaviour of the JUNE model. For example, we can examine the coefficients $b_{ij}$ of the linear terms $g_{ij}(x_{A_{i}})=x_{A_{i}}^{j}$ for the inputs featuring in equation (2.1), to gain insight into the effect each input has on each individual output. Estimates of these are shown in figure 3 for the total deaths in England outputs, where i index therefore cycles through just the various time points: $i\in \{\text{`}\mathrm{Total}\;\mathrm{Deaths}\text{'},\text{`}\mathrm{England}\text{'},t_{1}^{\prime },t_{2}^{\prime },\ldots \}$ with each time point, labelled on the x-axis, giving rise to a single vertical strip in the plot corresponding to a single emulator (note that a finer temporal resolution $\left\{t_{1}^{\prime },t_{2}^{\prime },\ldots \right\}$ is used here for added detail, while far fewer time points are used in the HM). Conversely, j labels the active input $x_{A_{i}}^{j}$ in question as given on the y-axis. Here, red/blue represents positive/negative dependencies $b_{ij}$, respectively, standardized as proportions of the largest coefficient of that output. We see strong anticipated contributions from $\beta_{\mathrm{company}}$, $\beta_{\mathrm{school}}$ and $\beta_{\mathrm{household}}$ in the first wave of the pandemic from March to May, and more modest effects from $M_{\mathrm{social}\;\mathrm{distancing}\;\mathrm{beta}\;\mathrm{factor}}$ and $\beta_{\mathrm{grocery}}$ throughout the summer period. The sensitivities of $\beta_{\mathrm{school}}$ and $\beta_{\mathrm{household}}$ change to negative (blue) by May, as in many of these uncalibrated runs, herd immunity has been reached, and hence increasing $\beta_{\mathrm{school}}$ will decrease deaths (as they will be brought forward in time). Note that the parameters $\beta_{\mathrm{householdvisits}}$ and $\beta_{\mathrm{care}\;\mathrm{visits}}$ are not included in figure 3 as they were only implemented prior to the second iteration of runs, but were included in the subsequent full history match by suitable inflation of the iteration 1 emulator uncertainties [20]. More insight can be gained from full emulator sensitivity analysis [50].

### (c) Iterative history matching

**Figure 5.**
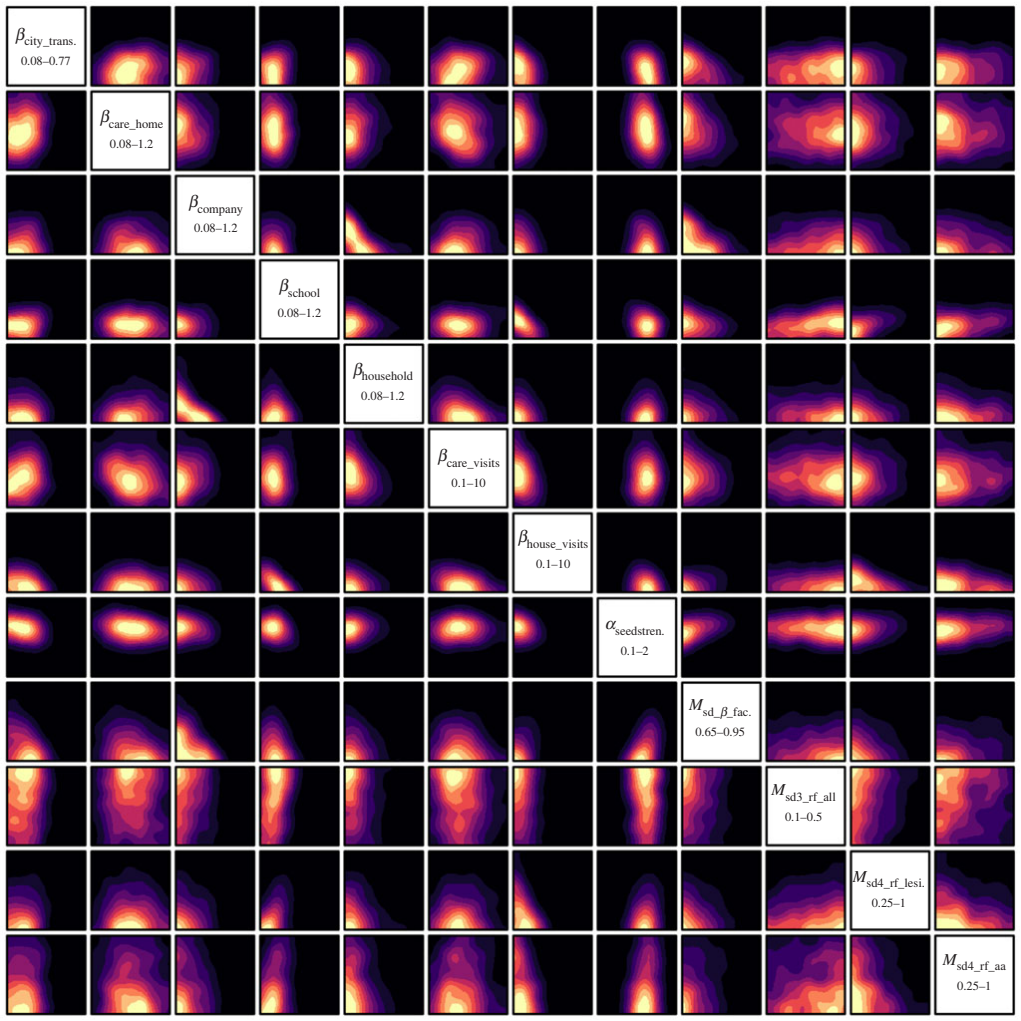
The optical depth $\rho (x^{\prime })$ of various two-dimensional projections of the full 20-dimensional non-implausible region $X_{5}$ found after the 5th iteration. The 12 most constrained inputs are show, labelled on the diagonal (the remaining eight inputs were relatively unconstrained). The colour scales are standardized and linear in depth, with yellow showing maximum depth for that projection and purple/black showing minimum/zero depth. This region corresponds to the red runs in figure 4. (Online version in colour.)

We now employ the history matching framework from §2c, iteratively removing parameter space based on current implausibility measures, and performing batches/iterations of further runs. Initially, in iterations 1 and 2, only the hospital and total deaths for England up to the end of May 2020 were included in the HM, to rule out the more exotic regions of parameter space, while for iterations 3–5, all the seven regions of England were also included and the time period extended to the end of August 2020. Figure 4 shows the outputs from iterations 1, 3 and 5 for total deaths in England as the purple, yellow and red lines, respectively. As the iterations proceed, the emulators become more accurate, we learn more about the global parameter space, and hence, the runs approach the observed data, yielding reasonable matches across the first COVID-19 wave. By iteration 5, the majority of emulators attained the accuracy required for the stopping criteria in the HM algorithm. The region $X_{5}$ of 20-dimensional parameter space deemed non-implausible at iteration 5 is shown in figure 5 as a collection of two-dimensional *optical depth plots,* which simply show the depth in the remaining 18 dimensions of the non-implausible region (see [4]). The optical depth $\rho (x^{\prime })$ is defined for each point $x^{\prime }$ in the two-dimensional space shown in each individual plot panel as follows:

3.4 $$\rho (x^{\prime })=\frac{V_{18}\{x\in X_{5}\;|\;x^{\prime }\;\text{fixed}\}}{V_{18}\{x\in X_{0}\;|\;x^{\prime }\;\text{fixed}\}},$$

where $V_{18}\{.\}$ denotes the 18-dimensional volume of the remaining space. $\rho (x^{\prime })$ can therefore show where large or small amounts of non-implausible points can be found, conditioned on $x^{\prime }$, providing further insight into the structure of $X_{5}$. Figure 5 gives insights into the constraints imposed on the parameters by the death data and corresponding uncertainty specification. For example, we see that we learn a lot about certain influential parameters such as $\beta_{\mathrm{school}}$, which are fairly well constrained, while others such as $\beta_{\mathrm{care}\;\mathrm{home}}$ can take a wider range of values. Provisional investigations suggest we can further constrain $\beta_{\mathrm{care}\;\mathrm{home}}$ by adding deaths in care home settings to the calibration outputs. We also see interesting relationships between pairs of parameters, e.g. the reciprocal relations between $\beta_{\mathrm{company}}$ vs. $\beta_{\mathrm{household}}$, suggesting one or other can be high, but not both. We see similar relations between $\beta_{\mathrm{company}}$ vs. $M_{\mathrm{social}\;\mathrm{distancing}}\;\beta \;\mathrm{factor}$. However, one should be aware that the actual constraints imposed are higher dimensional in nature and cannot be fully represented by such two-dimensional plots, but that they can be explored further e.g. by examining the eigenstructure of $X_{5}$, as done in ref. [51]. Note that in using HM in this way, we do not seek to probabilize the non-implausible region as in a full Bayesian calibration, but we could go on to do this (e.g. by routing the emulators through an MCMC algorithm) if desired, but the additional information gained may be in part an artefact of the particular additional distributional choices that such an analysis requires, which may impact robustness and predictive accuracy.

While performing a full global exploration of the input parameter space is of course preferable, it is sometimes useful to perform a fast ‘look-ahead’ stage to check if such an expensive model is capable of fitting the next period of observed data, or whether model improvements are required. Figure 4 also shows the results of such an exercise, where we took eight runs with acceptable matches to the death data up to the end of August, and performed small 30-point five-dimensional Latin hypercube designs for each of the eight cases up until December 2020, now varying only five additional parameters relevant to the second COVID-19 wave (social distancing for schools, leisure, and non-leisure activities, November lockdown and B.1.1.7 variant infectiousness), the output of which is given by the green lines. One iteration of HM was performed to reduce the five-dimensional parameter space in each case, and a new set of runs designed, which are shown as blue lines in figure 4. We see reasonable matches to the first part of the second COVID-19 wave, with perhaps a late take-off in early September, and a partial overshoot in November to December, suggesting that JUNE may well provide acceptable matches after a full HM.

**Figure 6.**
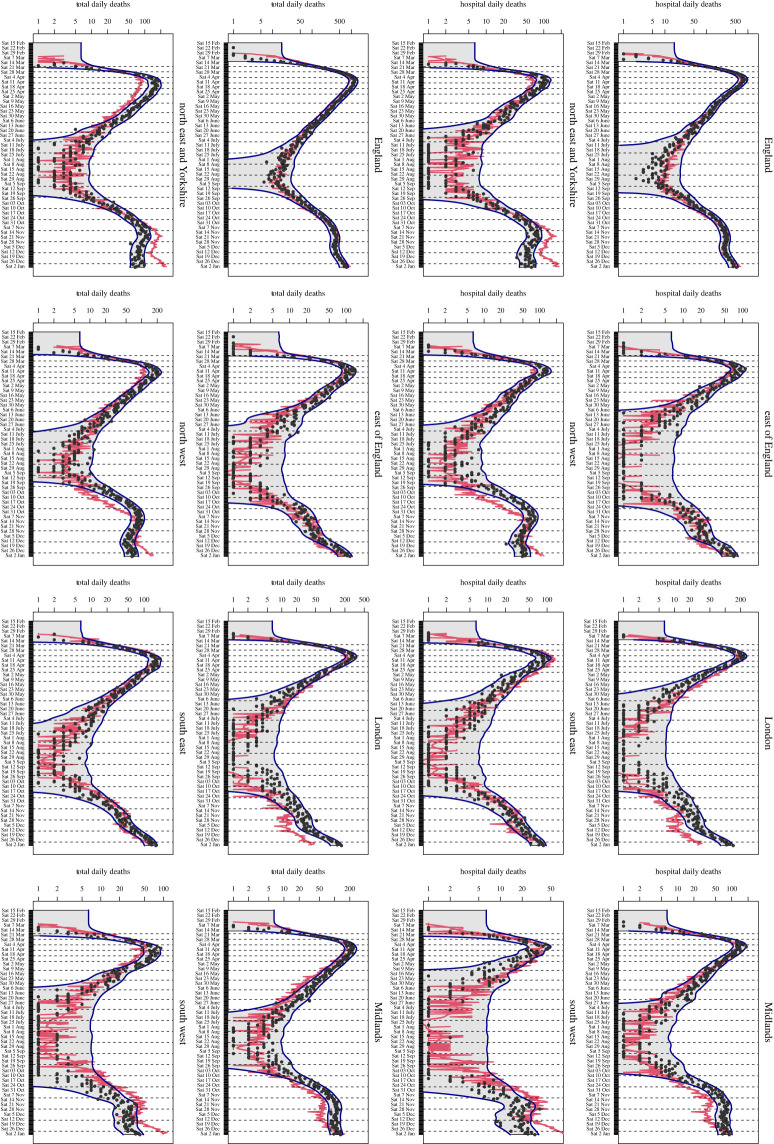
A single JUNE run (red lines), from the second exploratory iteration (i.e. one of the blue lines in figure 4). The panels show hospital deaths (rows 1 and 2, viewed in landscape) and total deaths (rows 3 and 4, viewed in landscape) for England and the seven regions, as given in the plot titles. The black points give the (unsmoothed) death data, and the combined uncertainties due to $\sigma_{e}$ and $\sigma_{\epsilon }$ are shown as the blue lines. (Online version in colour.)

To give more detail, figure 6 shows a single unsmoothed run (red lines), from this final batch, but now for hospital deaths and total deaths for England and all seven regions, and shows the sort of quality of matches we are seeing so far. The black points give the (unsmoothed) death data and the combined uncertainties due to $\sigma_{e}$ and $\sigma_{\epsilon }$ shown as the blue lines. The fact that JUNE matches several regions simultaneously, at least over the first wave, without resorting to any region specific parameters, suggests that geographical variations in the relative importance in different types of interaction drove/affected the different epidemic curves in those regions. Further, more detailed investigation to confirm this is of course required. We leave the extension to 2021 and beyond to the future work, as this requires the complex behavioural and partial restrictions (on travel, visiting relatives, etc.) imposed over the December 2020 Christmas period (the ‘cancelled Christmas’) and the January to April 2021 lockdown and subsequent staggered release to be implemented and tested, possibly requiring additional time-dependent parameters, which is the ongoing work (although we note that vaccines and multiple variants have already been implemented in JUNE). We also note that the process of using complex models combined with emulators and appropriate uncertainties to make realistic predictions over such periods is a substantive UQ topic in its own right, which deserves separate treatment [52].

### (d) Discussion

Models such as JUNE, with its high level of demographic and spatial granularity, may become important tools to aid local and national decision makers. However, to fully exploit the nuances of such complex and expensive ABMs, efficient and comprehensive calibration methods are required. We demonstrated the emulation of JUNE, providing insight into the model structure, and employed HM to identify the region of parameter space yielding reasonable matches to national and regional level hospital and total death data for the first COVID-19 wave. Such techniques form an essential tool for the future use of complex epidemiological ABMs, expanding our capabilities to combine detailed models with rigorous UQ. The ability to perform global exploration of the parameter spaces of expensive models of this form and to embed this within a broader UQ framework is vital for making predictions with realistic uncertainty statements and hence vital for subsequent decision support.

## 4. Outlook/future directions

Our work represents an important step towards the full exploitation of highly granular and detailed ABMs in health settings and elsewhere, harnessing the full depth of their simulations in providing high-quality understanding of critical dynamics and robust quantitative projections for improved decision support.

The next steps in this project are to include further outputs of interest within the HM for JUNE, (hospitalizations, case rates, age categories, etc.) and to examine smaller geographic regions, in which the stochasticity of JUNE will become more pronounced, compared to the national/regional level where it is somewhat subdominant. This will require more sophisticated emulator strategies [11], and if we are interested in detailed spatial predictions, will require the updating of the JUNE state vector using UQ style data-augmentation techniques [53]. Beyond this, these UQ methods are currently being incorporated wherever JUNE is being employed e.g. by the UN for Cox’s Bazaar [27], a refugee camp in Bangladesh, and for Rhineland-Palatinate [28], one of Germany’s federal states.

In addition, we plan to use the model to investigate in more detail social imbalances in COVID-19 attack rates and infection-fatality ratios, which are relatively easy to trace in a model such as JUNE. Supplementing the model with the elaborate UQ techniques will allow us to identify, in more detail and with increased certainty, important correlations between socio-economic markers of the population and the infection dynamics and outcomes.

## Data Availability

A full open source code base and implementation examples are available through github: https://github.com/IDAS-Durham/JUNE; pypi: https://pypi.org/project/june/. The history matching and emulation methods are available in the ‘hmer’; R package v1.0 (available from CRAN). The version of JUNE used for this work was v1.0 [54].

## Footnotes

1 The English and Welsh census distinguishes between children, young (dependent) and adults, such as university students, independent adults and old adults, and classifies households according to the respective numbers in them.

2 As an example consider the number of contact children have with adults in schools. Clearly the number of contacts of average adults with children in schools is much less than the number of contacts adult teachers have with the children, necessitating a renormalization of the number of contacts by the proportion of teachers in the overall adult population.

3 In other population settings, for example in refugee camps, age and sex do not constitute good proxies for the overall health of an individual, and comorbidities need to be explicitly factored in, giving rise to significant additional assumptions and uncertainties.
